# Dobbs and the future of health data privacy for patients and healthcare organizations

**DOI:** 10.1093/jamia/ocac155

**Published:** 2022-10-04

**Authors:** Ellen Wright Clayton, Peter J Embí, Bradley A Malin

**Affiliations:** Law School, Vanderbilt University, Nashville, Tennessee, USA; Department of Health Policy, Vanderbilt University Medical Center, Nashville, Tennessee, USA; Department of Pediatrics, Vanderbilt University Medical Center, Nashville, Tennessee, USA; Department of Biomedical Informatics, Vanderbilt University Medical Center, Nashville, Tennessee, USA; Department of Medicine, Vanderbilt University Medical Center, Nashville, Tennessee, USA; Department of Biomedical Informatics, Vanderbilt University Medical Center, Nashville, Tennessee, USA; Department of Biostatistics, Vanderbilt University Medical Center, Nashville, Tennessee, USA; Department of Computer Science, Vanderbilt University, Nashville, Tennessee, USA

**Keywords:** electronic medical records, consumer informatics, privacy

## Abstract

The Supreme Court recently overturned settled case law that affirmed a pregnant individual’s Constitutional right to an abortion. While many states will commit to protect this right, a large number of others have enacted laws that limit or outright ban abortion within their borders. Additional efforts are underway to prevent pregnant individuals from seeking care outside their home state. These changes have significant implications for delivery of healthcare as well as for patient-provider confidentiality. In particular, these laws will influence how information is documented in and accessed via electronic health records and how personal health applications are utilized in the consumer domain. We discuss how these changes may lead to confusion and conflict regarding use of health information, both within and across state lines, why current health information security practices may need to be reconsidered, and what policy options may be possible to protect individuals’ health information.

In *Dobbs v. Jackson Women’s Health Org,*^1^ the Supreme Court struck down the right to abortion previously guaranteed by the United States Constitution, holding that regulation of abortion is a matter for states to decide. Many states will continue to protect the right of pregnant individuals (referred to hereinafter as women) to make these essential healthcare decisions,^2^ but 22 states have already enacted laws that severely limit or ban abortion altogether, although their exact provisions vary from state to state.^3^ It is evident that other states will soon follow.^4^ Many of these laws impose severe criminal penalties on clinicians who provide abortions, and some extend penalties to people who help women who seek to terminate pregnancies. Although the laws in some states (eg, Texas) explicitly provide that the pregnant woman herself is excluded from these criminal penalties, other states are ambiguous on this point. Some people, including elected officials,^5^ are calling explicitly for preventing women from leaving their home state to terminate a pregnancy and prosecuting women who have abortions no matter where they obtain them. Texas famously passed a law^6^ that allows any person to bring a civil action for damages against anyone who helps a woman obtain an abortion, a model that is already being considered in other states. Prosecutors have already sought in some cases to convict women who sought to self-induce abortion,^7^ suggesting that longstanding practices of prosecuting pregnant women will likely increase.^8^^,^^9^ Physicians and health systems are also at risk at being investigated and intimidated even in states that still allow abortion, as demonstrated by the recent case involving a 10-year-old rape victim from Ohio who sought care in Indiana, prior to passage of that state’s near total ban.^10^^,^^11^ In such cases, the search for evidence of potential legal violations via requests for medical records and related health care information are common. As new laws take effect, we can anticipate that health care providers and covered entities will soon experience a conflict between their obligations to produce health information when compelled by law and their longstanding obligations to protect physician-patient confidentiality and prevent inappropriate access to protected health information (PHI) that could be used to intimidate and prosecute patients and health practitioners. Informaticians, like other healthcare professionals, are bound by a code of ethics that requires clear understanding of their obligations to patients and the public as well as the provisions of the new laws.^12^

Notably, various threats to privacy can arise within the healthcare organization(s) where a woman seeks care. The Health Insurance Portability and Accountability Act of 1996 (HIPAA) requires covered entities, including hospitals, clinicians’ offices, and their business associates, to protect personal health information in a wide range of settings.^13^ Yet, within healthcare organizations, the modern electronic health record (EHR) system is designed to be widely accessible by employees charged with facilitating the delivery of health care, as well as for payment, operations, and to ensure safety and quality. While most healthcare organizations strive to limit access on a “need to know” basis, including policies and procedures to discourage inappropriate access to records, such limitations can be difficult to realize in practice.^14^^,^^15^ Instead, it is common for healthcare organizations to allow reasonably broad EHR access so employees can meet the institution’s broader health care goals. Rather than apply fine-grained access controls from the outset, many organizations instead work to instill a culture of information protection through employee training and seek to deter illicit behavior by monitoring for and performing retrospective audits for suspicious use of EHRs.^16^ Moreover, when an employee violates a healthcare organization’s acceptable use policy, various disciplinary actions could be applied, ranging from retraining for minor infractions to loss of employment for serious violations of institutional policy. Even so, such practices may not always suffice to prevent employees from reporting medical care they find morally objectionable.

Shortly after *Dobbs* was announced, the Office for Civil Rights (OCR) of the U.S. Department of Health and Human Services issued guidance about HIPAA’s protection of information about reproductive health care which makes clear that these provisions forbid such disclosures on pain of federal penalties except as “required by law.” In the guidance, OCR provided several examples that make clear its narrow interpretation of this exception, language included in Table 1.

**Table 1.** A reproduction of the guidance issued by the Office for Civil Rights regarding reproductive rights (sans internal citations).^19^

Disclosures required by law
The Privacy Rule permits but *does not require* covered entities to disclose PHI about an individual, without the individual’s authorization, when such disclosure is required by another law and the disclosure complies with the requirements of the other law. This permission to disclose PHI as “required by law” is limited to “a mandate contained in law that compels an entity to make a use or disclosure of PHI and that is enforceable in a court of law.” Further, where a disclosure is required by law, the disclosure is limited to the relevant requirements of such law. Disclosures of PHI that do not meet the “required by law” definition in the HIPAA Rules, or that exceed what is required by such law, do not qualify as permissible disclosures.
Example:
An individual goes to a hospital emergency department while experiencing complications related to a miscarriage during the tenth week of pregnancy. A hospital workforce member suspects the individual of having taken medication to end their pregnancy. State or other law prohibits abortion after 6 weeks of pregnancy but does not require the hospital to report individuals to law enforcement. Where state law does not *expressly require such reporting*, the Privacy Rule would *not* permit a disclosure to law enforcement under the “required by law” permission. Therefore, such a disclosure would be impermissible and constitute a breach of unsecured PHI requiring notification to HHS and the individual affected.
Disclosures for law enforcement purposes
The Privacy Rule permits but *does not require* covered entities to disclose PHI about an individual for law enforcement purposes “pursuant to process and as otherwise required by law”, under certain conditions. For example, a covered entity may respond to a law enforcement request made through such legal processes as a court order or court-ordered warrant, or a subpoena or summons, by disclosing only the requested PHI, provided that all of the conditions specified in the Privacy Rule for permissible law enforcement disclosures are met.
In the absence of a mandate enforceable in a court of law, the Privacy Rule’s permission to disclose PHI for law enforcement purposes does not permit a disclosure to law enforcement where a hospital or other health care provider’s workforce member chose to report an individual’s abortion or other reproductive health care. That is true whether the workforce member initiated the disclosure to law enforcement or others or the workforce member disclosed PHI at the request of law enforcement. This is because, generally, state laws *do not* require doctors or other health care providers to report an individual who self-managed the loss of a pregnancy to law enforcement. Also, state fetal homicide laws generally do not penalize the pregnant individual, and “appellate courts have overwhelmingly rejected efforts to use existing criminal and civil laws intended for other purposes (eg, to protect children) as the basis for arresting, detaining, or forcing interventions on pregnant” individuals.
Examples:
• A law enforcement official goes to a reproductive health care clinic and requests records of abortions performed at the clinic. If the request is not accompanied by a court order or other mandate enforceable in a court of law, the Privacy Rule would *not* permit the clinic to disclose PHI in response to the request. Therefore, such a disclosure would be impermissible and constitute a breach of unsecured PHI requiring notification to HHS and the individual affected.
• A law enforcement official presents a reproductive health care clinic with a court order requiring the clinic to produce PHI about an individual who has obtained an abortion. Because a court order is enforceable in a court of law, the Privacy Rule would permit *but not require* the clinic to disclose the requested PHI. The clinic may disclose *only* the PHI expressly authorized by the court order.
Disclosures to avert a serious threat to health or safety
The Privacy Rule permits but *does not require* a covered entity, consistent with applicable law and standards of ethical conduct, to disclose PHI if the covered entity, in good faith, believes the use or disclosure is necessary to prevent or lessen a serious and imminent threat to the health or safety of a person or the public, and the disclosure is to a person or persons who are reasonably able to prevent or lessen the threat. According to major professional societies, including the American Medical Association and American College of Obstetricians and Gynecologists, it would be inconsistent with professional standards of ethical conduct to make such a disclosure of PHI to law enforcement or others regarding an individual’s interest, intent, or prior experience with reproductive health care.
Example:
A pregnant individual in a state that bans abortion informs their health care provider that they intend to seek an abortion in another state where abortion is legal. The provider wants to report the statement to law enforcement to attempt to prevent the abortion from taking place. However, the Privacy Rule would *not* permit this disclosure of PHI to law enforcement under this permission for several reasons, including: A statement indicating an individual’s intent to get a legal abortion, or any other care tied to pregnancy loss, ectopic pregnancy, or other complications related to or involving a pregnancy does not qualify as a “serious and imminent threat to the health or safety of a person or the public”. It generally would be inconsistent with professional ethical standards as it compromises the integrity of the patient-physician relationship and may increase the risk of harm to the individual.
Therefore, such a disclosure would be impermissible and constitute a breach of unsecured PHI requiring notification to HHS and the individual affected.

Abbreviations: HIPAA: Health Insurance Portability and Accountability Act of 1996; PHI: protected health information.

HIPAA’s protections, while broad, are not absolute, and the laws governing abortion care across the United States are often unclear, evolving, and conflict among states. As such, stakeholders including patients, clinicians, informaticians, health systems, and their business associates now face challenges regarding the routine collection and use of health information. We can already identify and anticipate a variety of potential threats to stakeholders arising from access to health information that heretofore did not portend legal liability. In the sections that follow, we discuss several key problem scenarios and recommend specific mitigation measures to help protect the information privacy rights of individuals as well as the sanctity of the clinician-patient relationship. We have summarized these scenarios in Table 2 and illustrate how they progress from situations that take place when patient data are stored within a healthcare institution (eg, an employee in a healthcare organization misuses an EHR) to those that occur when data are moved outside of a healthcare organization (eg, when a patient uploads data to mobile health app). We further suggest opportunities for risk mitigation, such as how organizations could reiterate the need to maintain patient confidentiality and use EHRs in a manner that is consistent with internal policy.

**Table 2.** Various ways in which information about an individual’s abortion could be subject to privacy intrusions and potential ways to resolve such threats

Scenario	Threats	Example	Opportunities for resolution
Employee in a Healthcare Organization (HCO) Misuses EHR	Employee accesses PHI without justification	Clinician searches for information about a patient considering an abortion and reads the medical record of a patient for whom they are not providing care	Increase access control granularity and auditing to ensure access on a need-to-know basis (eg, flag records of possible interest) Reinforce education for health professionals about patient privacy
Healthcare Organization Employee Shares Information	Employee accesses PHI and alerts law enforcement of possible antiabortion law violation	Clinician reads note in patient record for complications of possible abortion, no matter where it occurred, and alerts authorities	Educate employees that PHI is protected no matter where the care occurred and that decisions to disclose it should be made by the institution and not the employee
Healthcare Organization Shares Information	HIPAA requires protection of PHI, but there are exceptions as “required by law”	A prosecutor makes a request for EHR records given suspicion of abortion care	Healthcare organizations should follow guidance from the Office for Civil at the U.S. Department of Health and Human Services and not comply with request without a specific statute or presentation of a subpoena, warrant, or court order (see Table 1)
Business Associate (BA) of Healthcare Organization Shares Information	Business associate shares data more readily than health system due to different interpretation and internal policies	A prosecutor makes a request for EHR records to BA rather than healthcare system given suspicion of abortion care	HCO should enter into data use agreements (DUAs) with business associates requiring them to comply with institutional policy and notify HCO before release BA should follow guidance from the Office for Civil at the U.S. Department of Health and Human Services and not comply with request without a specific statute, or presentation of a subpoena, warrant, or court order (see Table 1)
Patient Downloads Information	Patients download data from EHR and share with third parties that are not bound by HIPAA	An Individual shares data from their healthcare provider’s EHR to their smart-phone with third party apps and they have “consented” to allow further sharing	Encourage healthcare organizations to inform patients that following sharing such data is no longer covered by HIPAA Expand definition of health information
Consumer Uses Application to Document Health Information	Data from personal apps are made available to or accessed by law enforcement to screen for and/or serve as evidence of failed pregnancy	A woman keeps track of her period using a mobile application in which the terms of service do not provide limitations on resharing	Congress could consider the expansion of the definition of protected health information under HIPAA Congress could pass new laws to protect privacy rights more broadly

Abbreviations: EHR: electronic health record; HIPAA: Health Insurance Portability and Accountability Act of 1996; PHI: protected health information.

Let us consider the following hypothetical—a woman in a state that forbids elective abortion, like Tennessee, travels to a more permissive state at the suggestion of her healthcare provider to obtain the procedure. She subsequently returns to Tennessee, only then to suffer a complication from the procedure. While this clinical presentation would likely resemble a miscarriage (ie, a spontaneous abortion), EHR-based documentation could be created that confirms the presentation as the result of an induced abortion. Information may also be retrieved electronically from the EHR system in the other state where she sought care, and would likely contain information about her abortion in that setting. In the face of such a threat, it is possible that organizations in states where abortion is illegal may choose not to access certain records of a patient’s medical history, thus compromising her care and leading to further health inequities. Even if not available, if the patient shares her medical history, new documentation could be created in the local EHR system about her decision and efforts to pursue abortion elsewhere and subsequent care for her complication. No matter how it finds its way into the local EHR, information about such clinical encounters could then be discovered, including by someone with access who may be under the impression that the patient’s or clinician’s actions are inconsistent with the law.

The potential threats to the care of pregnant women and to their clinicians extend well beyond abortion. A particularly striking example is that women are increasingly at risk of receiving inadequate care following miscarriages, which are common and can be incomplete, meaning that pregnancy-related tissues (eg, fetal and placental tissues) are not completely expelled from the uterus. Removing the remains of the failed pregnancy is often essential to protect the health and life of the woman, but a growing number of reports that indicate providers are hesitating to provide such care^17^ because they fear being implicated in abortion. Additionally, women who miscarry increasingly report that clinicians suspect them of seeking or having attempted abortions, further compounding their stress, which if documented in the EHR could expose them to condemnation and their prior providers to criminal prosecution.^17^^,^^18^

One mitigation consideration that may seem obvious is that some may simply wish not to document certain health events in the EHR. However, this course of action may not always be practical, clinically safe, or legally appropriate. For instance, accountability issues may limit the organization’s ability to omit health care information, since the organization may need to provide documentation about why and how care was administered in the event of an adverse event. Moreover, if the patient is relying upon health insurance to cover some of their care, detailed documentation may be necessary for reimbursement. While the latter situation could possibly be addressed by developing various types of abortion-related events grouped into more generic descriptions of care for failed pregnancies, such a practice and new documentation standards would need to be agreed upon by providers and payers, and it would not address the need for appropriate clinical documentation.

To minimize the number of employees with access to such information, organizations could consider creating a segmented patient record where pregnancy-related health events are separated from other aspects of care. There is precedent for this; for instance, psychiatric and psychotherapy documents are provided a higher level of protection than other aspects of the medical record, often with break-the-glass capabilities that trigger review if unauthorized users attempt access. Still, segmentation of routine health information can be problematic in numerous ways including, inaccurate record categorization, challenges managing access rights, and especially because any restrictions to information can lead to incorrect medical decision making and suboptimal care.^20^ Further, it has been illustrated that segmentation amplifies inequities for certain types of care (eg, addiction treatment),^21^ particularly for patients who are poor or minorities, the same groups who are particularly at risk of harm by abortion bans. Nonetheless, concerns that sensitive health information may be revealed by others could lead clinicians to consider changes to documentation practices, despite possible impacts on care. At a minimum, providers and their pregnant patients should be informed about the potential implications of having abortion care documented in EHRs, and ideally documentation practices should be standardized to enable care across sites while mitigating threats to liberty.

These scenarios illustrate the tension between confidentiality in healthcare as defined and (somewhat) protected by HIPAA and antiabortion laws at the state level. However, HIPAA-governed information is not the only source of concern in the post-*Dobbs* era. Information about potential or actual pregnancy status and termination could also be captured or inferred from personally controlled environments, such as personal health records, mobile apps (eg, period trackers), or posts to social media.^22^ In addition, some patients download their medical records and upload them to such sites. In many ways, these sources are potentially even more problematic since such environments are outside of the oversight of HIPAA entirely.

Outside of HIPAA-covered entities, the patient is considered to be a general consumer, entitled only to the privacy protections service providers provide in a terms of service, privacy policy, or end user licensing agreement (EULA). Most consumers fail to read these agreements, and so are at the mercy of the service provider.^23^^,^^24^ This is a concern because recent estimates show that almost 90% of health apps collect user data.^25^ Moreover, service providers are generally free to change their privacy policies at will. Thus, if a service provider indicates that they retain the right to share data without the consumer’s consent, then (unless they do so in a manner that intentionally harms the consumer) they can likely do so without the consumer’s objection. Unlike the healthcare setting, here the Federal Trade Commission (FTC) oversees the relationship between consumers and service providers, and can only intervene, as specified by Section 5 of the FTC Act, for “unfair or deceptive acts or practices in or affecting commerce”.

More important, there are already numerous reports that women who seek or have abortions can be identified by examining the data they store in personal apps or by the information they seek.^26^ This is leading some entities to change the way they store data, although many of the major data holders and financial entities are not forthcoming about their practices despite this new risk to women.^27^

Some have suggested that this issue could be resolved by extending HIPAA to cover organizations that are neither covered entities nor business associates, proposing that the regulation cover any environment in which information about one’s health is communicated, such as app makers.^28^^,^^29^ Among various implications, however, this would require HIPAA to change the definition of healthcare as well as subject a number of organizations to onerous HIPAA compliance requirements. Another strategy is to provide broader protections for consumers more generally. The first wave of such efforts are state-level consumer data protection acts. To date, four states have enacted such laws, including California,^30^ Colorado,^31^ Virginia,^32^ and Utah,^33^ with many other states seemingly ready to follow suit. While these laws have limitations (eg, they typically only cover businesses that achieve a certain level of revenue) and vary in their applicability, they provide consumers with a greater level of control over how personal data are shared. Further, a bipartisan and bicameral bill, the American Data Privacy and Protection Act,^34^ was recently introduced into Congress, which aims to codify many of the principles established in the state data privacy laws. Enacting a federal statute would be far better than a state-by-state solution given the distributed nature of such information systems. It is unclear, however, if this or similar bills will be enacted.

Even as new state laws take effect and the legal landscape evolves following the *Dobbs* decision,^35^ clinicians and informatics professionals need to be mindful of the many laws and policies that protect patient information. In particular, it is imperative to recognize that those among us responsible for supporting and enabling health care and entrusted with the management of healthcare information are particularly bound by ancient obligations to protect the confidentiality of those who have entrusted us with their care, often at their most vulnerable moments.^12^

## AUTHOR CONTRIBUTIONS

All authors contributed equally to development and writing of this manuscript.

## CONFLICT OF INTEREST STATEMENT

None declared.

## Data Availability

There is no data associated with this manuscript.
