# Correction to: Dobbs and the future of health data privacy for patients and healthcare organizations

**DOI:** 10.1093/jamia/ocac183

**Published:** 2022-10-04

This is a correction to: Ellen Wright Clayton, Peter J Embí, Bradley A Malin, Dobbs and the future of health data privacy for patients and healthcare organizations, *Journal of the American Medical Informatics Association*, 2022; ocac155, https://doi.org/10.1093/jamia/ocac155

This notice serves to notify readers that a new and revised version of this paper has been published. During production of this manuscript the author’s original submission was inadvertently uploaded in place of the revised submission incorporating feedback from the reviewers. The correct version has now been published.

